# Calcium Sensing Receptor Inhibits Growth of Human Lung Adenocarcinoma Possibly via the GSK3β/Cyclin D1 Pathway

**DOI:** 10.3389/fcell.2020.00446

**Published:** 2020-06-25

**Authors:** Jiansha Li, Pu Liao, Kun Wang, Zhuangzhuang Miao, Rui Xiao, Liping Zhu, Qinghua Hu

**Affiliations:** ^1^Institute of Pathology, Tongji Hospital, Wuhan, China; ^2^Key Laboratory of Pulmonary Diseases of Ministry of Health of China, Wuhan, China; ^3^Department of Pathology, School of Basic Medicine, Tongji Medical College, Huazhong University of Science and Technology, Wuhan, China; ^4^Department of Pathology, Union Hospital, Wuhan, China; ^5^Department of Nephrology, Tongji Hospital, Wuhan, China; ^6^Department of Neurosurgery, Tongji Hospital, Wuhan, China; ^7^Department of Pathophysiology, School of Basic Medicine, Huazhong University of Science and Technology, Wuhan, China

**Keywords:** CaSR, lung adenocarcinoma, proliferation, GSK3β, Cyclin D1

## Abstract

The effect of calcium sensing receptor (CaSR) on tumor cell proliferation has been studied in several human cancers, and great discrepancies were found in different tumors. However, the role of CaSR in lung adenocarcinomas (LUADs) is not clear. Therefore, we investigated the function of CaSR on regulating the growth of human LUAD and its possible mechanism. The expression of CaSR protein and its relationship with pathological parameters were examined in paraffin sections from 51 LUAD patients, by immunohistochemistry. The results showed that CasR expression was negatively correlated with the Ki-67 index as well as the grade of malignancy in LUAD. Further, CaSR demonstrated an *in vitro* inhibitory effect on the proliferation of human LUAD A549 cells by regulating CaSR activity with agonist cinacalcet, antagonist NPS2143, or shRNA-CaSR transfection. Tumor xenograft models also verified the *in vivo* proliferation-inhibiting role of CaSR by subcutaneous injecting A549 cells into nude mice with or without changes of CaSR activity. Molecularly, Western blotting showed that CaSR positively regulated the activity of glycogen synthase kinase 3β (GSK3β), followed by the downregulation of Cyclin D1. We used the dominant negative mutant and the constitutively active mutant plasmid of GSK3β to alter GSK3β activity. Our functional experiments showed that the proliferation–inhibition of CaSR was suppressed by the inactivation of GSK3β and enhanced by the activation of GSK3β. These results suggested that CaSR played a proliferation-inhibiting role in LUAD, at least partially by regulating the GSK3β/Cyclin D1 pathway.

## Introduction

Lung cancer is the most fatal tumorous disease in the world (Siegel et al., 2019). Its most prevalent histological type, lung adenocarcinoma (LUAD), has shown high relative frequency and accounts for at least half of all cases of non-small cell lung carcinoma (Kumarakulasinghe et al., 2015; Travis et al., 2015). Despite significant advances in conventional surgery, chemotherapy, and radiotherapy, LUAD still has an unacceptable prognosis (Toloza et al., 2006; Bittner et al., 2014). Recent advances in the understanding of cancer genome alterations in LUAD have provided new treatment targets. In clinical practice, some LUAD patients with specific driver oncogene aberrations (such as EGFR and ALK) have already benefited from the precision medicine (Saito et al., 2016). However, there are still many patients who lack the well-known “druggable” driver oncogene aberrations (Saito et al., 2016) and suffer from poor prognosis. Therefore, exploiting novel molecular targets for LUAD is urgently needed.

Calcium sensing receptor (CaSR), a G-protein-coupled receptor (GPCR), first cloned and characterized by Brown et al. (1993), is sensitive to extracellular Ca^2+^ changes. In human cells, CaSR is ubiquitously observed and is involved in different signaling pathways (Gerbino and Colella, 2018). Some studies indicate that CaSR plays a critical role in either suppressing or promoting the progression of several human cancers. Xie et al. (2017) found that CaSR expression was elevated in gastric cancer specimens and it was considered as a correlate of the tumor progression as well as the poor survival of these patients. Their *in vitro* studies further manifested CaSR activation, which promoted tumor cell growth and migration through a Ca^2+^/AKT/β-catenin relay in gastric cancer cell lines (Xie et al., 2017). Similarly, highly expressed CaSR was responsible for promoting extracellular calcium on cell migration and proliferation in bone metastasizing renal cell carcinoma cells (Joeckel et al., 2014; Brenner et al., 2015). A latest review about CaSR and breast cancer illuminated that CaSR acts as an oncoprotein to stimulate cancer cell proliferation as well as skeletal metastases, and is therefore a new target for breast cancer with early-stage bone metastases (Das et al., 2020). In PC-3 prostate cancer cell line, the upregulation of CaSR induced by high [Ca^2+^]_out_ increased cell proliferation, while the silence of CaSR reduced cell proliferation, indicating the proliferation-promoting role of CaSR in prostate cancer (Bernichtein et al., 2017). Besides, in prostate, growing evidences indicate that the activation of CaSR may promote inflammation and eventually cancer development (Anract et al., 2019). In contrast, in the colon, the activation of CaSR is protective against inflammation and cancer (Iamartino et al., 2018). Consistently, in a clinical study with large sample sizes and long follow-up periods, Yang et al. (2018) found that CaSR-positive expression status was essential for the antineoplastic effect of calcium in colorectal cancer (Yang et al., 2018). Our previous work showed that CaSR activation reduced cell viability and induced apoptosis in endometrial cancer (Xin et al., 2018). These discrepancies of the roles of CaSR on tumor progression among different types of cancers imply that it might be tissue or disease specific. Up until now, little has been known about the effect of CaSR in LUAD. A recent clinical study demonstrated that CaSR protein expression was downregulated in LUAD tissue and the patients with a strong CaSR expression had longer overall survival (Wen et al., 2015). However, the role of CaSR in LUAD is still very unclear.

Therefore, we investigated the role of CaSR on LUAD growth regulation and its mechanism by using human LUAD specimens, A549 cells, and a tumor xenograft model. Our findings demonstrated that CaSR suppresses LUAD cell proliferation possibly by regulating the GSK3β/Cyclin D1 pathway.

## Materials and Methods

### Ethical Approval

The protocols in this work were ratified by the Ethics Committee of Tongji Medical College, HUST. Informed consent was obtained from the patients involved. All experiments were designed based on the required guidelines and regulations. We obtained approval for animal experiments involving BALB/c nude mice from the Institutional Animal Care and Use Committee and National Institutes of Health Guide for the Care and Use of Laboratory Animals.

### Tissue Specimen Collection

Formalin-fixed paraffin-embedded specimens of resected LUAD (2009.1–2011.12) were collected from 51 patients at Wuhan TongJi Hospital. Two pathologists confirmed each diagnosis and the grade of LUAD, according to 2015 WHO Classification of Lung Tumors. Before surgery, neither radiotherapy nor chemotherapy was applied to any of the patients.

### Cell Culture and Reagents

Human LUAD A549 cell line was purchased from the Cell Bank of Chinese Academy of Sciences (Shanghai, China). CaSR agonist cinacalcet (catalog number: SML2012-50MG) and antagonist NPS 2143 (catalog number: SML0362-25MG) were purchased from Sigma-Aldrich. We treated A549 cells with cinacalcet (0, 0.3, 1, 3, and 10 μM) for 90 min, or NPS 2143 (0, 0.025, 0.05, 0.1, and 1 μM) for 24 h.

### Plasmids and Gene Transfection

A549 cells were transfected with shRNA-CaSR plasmid and its empty control vector (Origene), separately, by Lipofectamine 2000 (Invitrogen, CA, United States) as previously described (Zhang et al., 2012). The nature of GSK3β plasmids used here has been verified by former work, S9A-GSK3β—constitutively active, and DN-GSK3β—dominant negative (Li et al., 2008). For cotransfections, each GSK3β plasmid was paired with the shRNA-CaSR plasmid at a molar ratio of 1:5 to ensure shRNA-CaSR would be co-expressed with GSK3β simultaneously. Transduction of shRNA-CaSR *in vivo* was performed as described in our previous study (Zhang et al., 2012).

### Cell Viability Assays

MTS colorimetric assay (Cell Titer, Promega Corporation, Madison, WI, United States) was adopted to decide the role of CaSR to modulate A549 cell growth. Briefly, A549 cells in 100 μl of culture medium with different treatments (cinacalcet, NPS 2143, or transfected with shRNA-CaSR, or transfected with shRNA-CaSR and GSK3β) were cultured in 96-well plates, approximately 2.5 × 10^3^ cells/well. At designated time points, after 90 min for cinacalcet treatment, or after 24 h in the presence of NPS 2143 treatment or plasmid transfections, we added 10 μl/well of CCK-8 solution and then incubated them at 37°C for additional 2 h. We determined the cell viability by reading the absorbance at 450 nm, with the reference wavelength at 600 nm. Control wells containing no cells were used for background absorbance. The relative cell viability (%) was calculated as: [A450 (treated) - A450 (blank)]/[A450 (control) - A450 (blank)].

### Western Blot

Whole cell lysates were obtained from different experimental A549 cells (cinacalcet treatment, NPS-2143 treatment, shRNA-CaSR transfection, shRNA-CaSR and GSK3β cotransfection, and no-treatment control). We performed three independent experiments with protein samples or preparations extracted separately. In each experiment, we used the same protein samples and run in different gels for different proteins due to their great disparity (GAPDH and CaSR) or great similarity (GAPDH, GSK3β, P-GSK3β, and Cyclin D1) in molecular weight. However, in each experiment, we performed gel electrophoresis as well as following protein transfer for GAPDH and the aim protein always under the same conditions including the same chamber, buffer systems, power supplies, and time duration. After equal amounts of proteins were loaded for Western blot, different primary antibodies were used for incubation. These antibodies included anti-CaSR, anti-P-GSK3β-ser9, anti-GSK3β, and anti-CyclinD1 (1:500, 1:500, 1:1000, and 1:150, respectively, CST, Beverly, MA, United States). Next, 1:5000 diluted HRP-linked antibodies (Santa Cruz, CA, United States) were employed for further incubation. An Amersham ECL chemiluminescent detection system was applied to develop blots. GAPDH served as the quantity control for protein loading.

### *In vivo* Experimental Protocol

Forty male BALB/c nude mice (4 weeks old) were bought from the Chinese Academy of Medical Sciences Institute of Experimental Animals (Beijing, China) to establish tumor xenograft models of LUAD. The mice were randomly divided into five groups of eight. Either untreated or treated A549 cells (4 × 10^7^) were subcutaneously inoculated into the axillary region of these experimental mice. The five groups were described as follows: (1) the blank control group injected with untreated A549; (2) the empty vector group injected with empty vector-transfected A549; (3) the shRNA-CaSR group injected with shRNA-CaSR-transfected A549; (4) the cinacalcet group injected with untreated A549 for later cinacalcet treatment; and (5) the NPS 2143 group injected with untreated A549 for later NPS 2143 treatment. One week after the injection, xenografts in all the groups had grown to a visible size of more than 0.5 cm in diameter. Then, for the last two groups, daily direct injections of cinacalcet (3 μM) or NPS 2143 (0.05 μM) into subcutaneous tumors were performed, correspondingly. After 14 days, all experimental mice were sacrificed. The subcutaneous tumors were removed for weight and volume measurements; the latter calculated as *v* = (length × width^2^)/2.

### Immunohistochemistry

A standard streptavidin–biotin–peroxidase complex method was selected to examine CaSR and Ki-67 expression in paraffin sections of LUAD. After the de-paraffinization and rehydration, a short period (about 15 min) of incubation within 3% H_2_O_2_ was adopted to block the background endogenous peroxidase activity. For antigen retrieval, we used a microwave oven to boil the slides in 10 mM citrate buffer (pH 6.0). After that, the specimens were shaken within sheep serum solutions at room temperature for 30 min to block non-specific antigens. Then, the anti-CaSR antibody and the anti-Ki-67 antibody (1:100, respectively, #ab 15580, Abcam) were chosen as the primary antibodies (overnight, 4°C). Samples only incubated with PBS served as the negative control.

The intensity of CaSR staining was evaluated semi-quantitatively, based on a four-point system: 0, 1, 2, and 3 (for none, faint, moderate and strong staining). Also, the extent of staining in the tumor cells was examined and scored from 0 to 4 (0, <5%; 1, 5–25%; 2, 26–50%; 3, 51–75%; and 4, >75%). The product of these two numbers was recorded as the final score of each LUAD section, ranging from 0 to 12 (Liu et al., 2018). These scores were used to determine the CaSR expression levels: 0–4, low (1+); 5–8, moderate (2+); and 9–12, high (3+).

### Statistical Analysis

Sigmaplot (version 12.5) was used for data analysis. The statistical values were reported as mean ± standard deviations. Multiple hypothesis methods, such as Spearman Rank Order Correlation and Pearson Product Moment Correlation, were adopted for correlation analysis. We used a one-way ANOVA followed by Fisher LSD method to determine the difference in the mean values between groups. Statistical significance was found for *p* < 0.05.

## Results

### The Expression of CaSR and Its Relationship With the Ki-67 Index as Well as the Grade of Malignancy in Human LUAD Specimens

**FIGURE 1:**
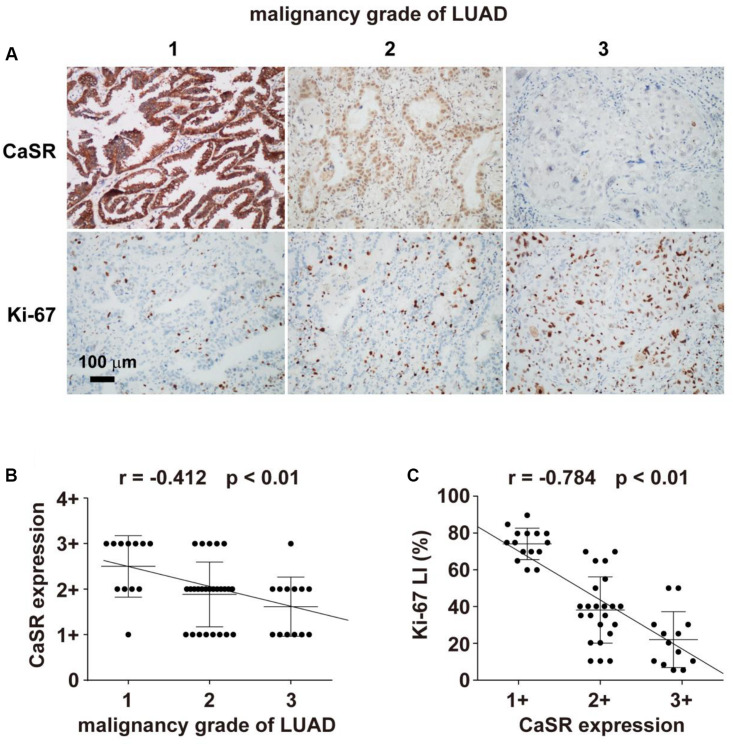
Immunohistochemical staining of CaSR and its correlation with malignancy grades of LUAD and Ki-67 LI. **(A)** Representative LUAD samples of grades 1, 2 and 3, respectively. (**A**, upper) Immunohistochemical staining of CaSR in LUAD (IHC × 400); (**A**, lower) Immunohistochemical staining of Ki-67 in LUAD (IHC × 400); **(B)** The correlation analysis between CaSR expression and malignancy grades of LUAD (Spearman Rank Order Correlation, *r* = −0.412, *p* < 0.01); **(C)** The correlation analysis between CaSR expression and Ki-67 LI (Pearson Product Moment Correlation, *r* = −0.784, *p* < 0.01).

Fifty-one cases of excised LUAD specimens were histologically divided into grade 1, grade 2, and grade 3, which corresponded to well, moderately, and poorly differentiated tumors. According to WHO Classification of Tumours of the Lung, Pleura, Thymus and Heart (4th edition, Travis et al., 2015), the grading system of LUAD we adopted here is based on the histological patterns, which have been shown to possess prognostic association. To be specific, in the current study, LUAD with lepidic predominant pattern is classified as grade 1 group (12 cases); LUAD with acinar, or cribriform, or papillary predominant pattern is included in the grade 2 group (26 cases); and LUAD with solid or micropapillary predominant pattern is determined as the grade 3 group (13 cases). The quantities and localizations of CaSR were determined by IHC. Positive staining of CaSR was detected in various subcellular locations. In grade 1 tumors, CaSR positive staining was usually observed in cell membrane and cytoplasm, while in many cases of grade 2 and grade 3 tumors, the positive staining of CaSR was also found in nucleus, in addition to cell membrane and cytoplasm. We found extensive and strong staining intensities of CaSR in normal bronchial and alveolar epithelia, as well as in most cases of grade 1 LUAD. When the tumor malignancy increased to grade 2 or 3, CaSR expression decreased, shown as the reduced positive scopes and staining intensity in Figure 1A. Correlation analysis demonstrated a negative relationship (*r* = -0.412, *p* < 0.01) between the CaSR expression and the malignancy grade of LUAD (Figure 1B). Oppositely, Ki-67 labeling index (Ki-67 LI) increased with an increasing malignancy grade of LUAD (Figure 1A). Furthermore, correlation analysis revealed a negative relationship (*r* = −0.784, *p* < 0.01) between Ki-67 LI and CaSR in LUAD (Figure 1C), indicating a potential proliferation-inhibiting role of CaSR.

### CaSR Played a Proliferation-Inhibiting Role in Human LUAD A549 Cells

**FIGURE 2:**
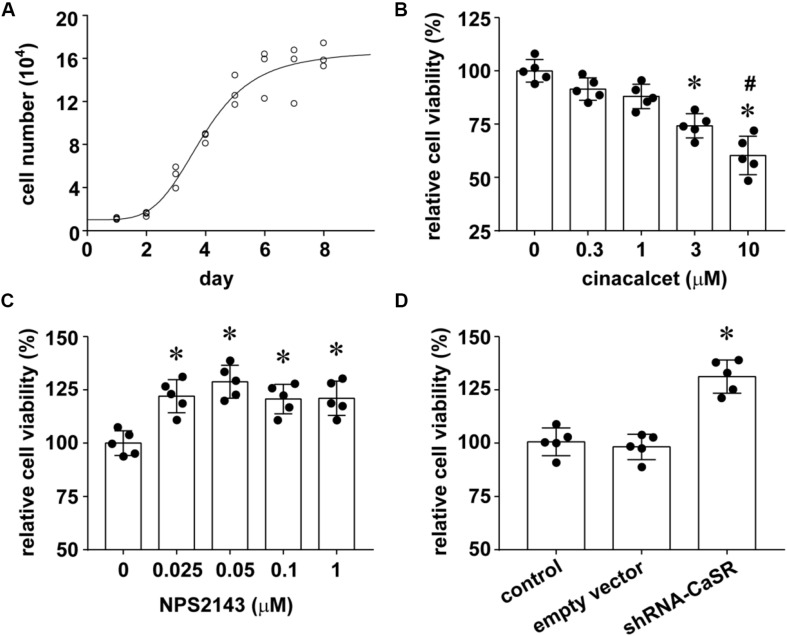
The effect of CaSR on A549 cell growth. **(A)** The growth curve of normal A549 cells; **(B)** CaSR agonist cinacalcet treatment (^∗^*p* < 0.05 vs. 0, 0.3, 1 μM, #*p* < 0.05 vs. 3 μM); **(C)** CaSR antagonist NPS2143 treatment (^∗^*p* < 0.05 vs. 0 μM); **(D)** shRNA-CaSR plasmid-transfected A549 cells (^∗^*p* < 0.05 vs. control and shRNA-vector). Significance was assessed using one-way ANOVA followed by Fisher LSD method. Error bars denote means ± standard deviations (SD) (*n* = 5).

To validate the possible proliferation-inhibiting function of CaSR in LUAD, we performed cell proliferation assay in human LUAD cell line A549. Based on the growth curve of A549 cells (Figure 2A), we chose day 3 (the time point at which the cells enter the logarithmic phase from the latent phase) to examine the proliferation of A549 cells with or without treatment of CaSR agonist cinacalcet, CaSR antagonist NPS 2143, or interference plasmid shRNA-CaSR.

The concentrations and time durations of cinacalcet and NPS 2143 treatment we adopted here have proven to be effective and safe in many documents with many cell types. In Flp-In HEK293-TREx c-myc-CaSR cells, Davey et al. (2012) performed time-course experiment and concentration-response assay for cinacalcet (0, 0.01, 0.03, 0.1, 0.3, and 1 μM) and (0, 0.01, 0.03, 0.1, 0.3, 1 and 3 μM) treatment, and demonstrated CaSR activation of cinacalcet and CaSR inhibition of NPS 2143 in a concentration-dependent manner with a time duration longer than 20 min. Consistently, Letz et al. (2014) indicated the inhibition effect of NPS 2143 (0.3 and 1 μM) on CaSR activity in HEK293 T cells. In a study about mouse brain bEND.3 endothelial cells, the authors found 24 h of 1 μM NPS 2143 treatment inhibited CaSR activity, while shorter time duration produced variable effects (Chen et al., 2019). In another research about pharmacologic modulation of CaSR in hematopoietic stem cell, the authors showed that cinacalcet treatment (2.5 μM, 90 min) enhances CaSR signaling without producing cellular toxicity (Lam et al., 2011). Our results demonstrated that the proliferation of A549 cells was inhibited by CaSR agonist cinacalcet treatment for 90 min in a dose-dependent manner. We found that 3 and 10 μM cinacalcet significantly inhibits A549 cell proliferation (^∗^*p* < 0.05). The result suggested increasing CaSR viability inhibited A549 cell proliferation (Figure 2B). On the other hand, we treated A549 cells with CaSR antagonist NPS2143 for 24 h and observed the changes in cell proliferation. Compared to the untreated group, all NPS2143 treatments (0.025, 0.05, 0.1, and 1 μM) statistically promoted A549 cell growth (Figure 2C), supporting our conclusion that CaSR inhibits A549 cell proliferation.

To further enhance the above conclusion, we decreased the expression of CaSR by shRNA-mediated knockdown and investigated its impact on the growth of A549 cells. The A549 proliferation profile of the shRNA-vector-transfected group resembled the blank control group. However, the shRNA-CaSR-transfected cells with an incubation of 24 h in 96-well plates displayed a dramatically increased rate of proliferating (^∗^*p* < 0.05; Figure 2D).

In summary, increasing CaSR activity inhibited A549 cell proliferation, while decreasing either CaSR activity or CaSR protein expression promoted A549 cell proliferation. These data lead to the conclusion that CaSR played a proliferation-inhibiting role in A549 cells *in vitro*.

### CaSR Played a Proliferation-Inhibiting Role in Implanted LUAD in Nude Mice

**FIGURE 3:**
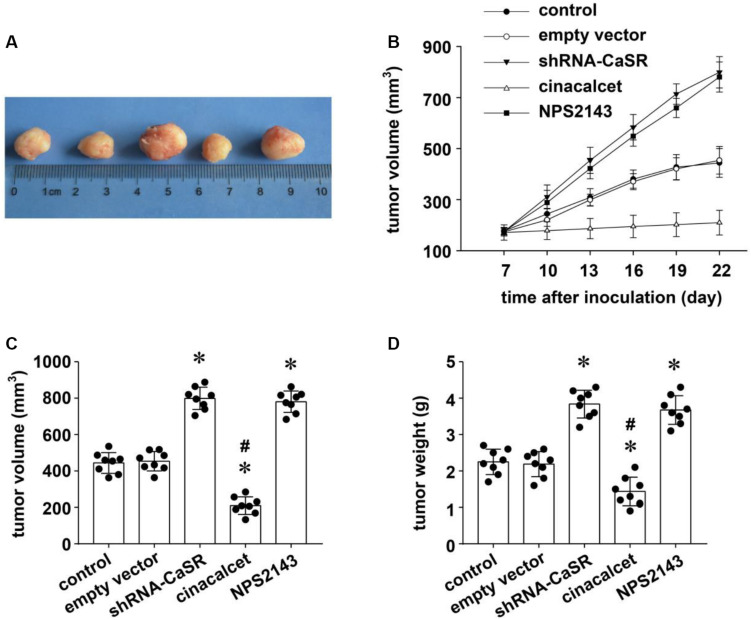
The effect of CaSR on the proliferation of LUAD *in vivo* in A549-implanted tumor xenograft models in nude mice. Five random groups of BALB/c nude mice were implanted with the same quantity of empty vector-transfected A549 (the empty vector group), shRNA-CaSR-transfected A549 (the shRNA-CaSR group), and untreated A549 for the remaining three groups. Seven days later, 2 groups of mice that received untreated A549 were then treated with intratumoral injection of either cinacalcet (the cinacalcet group) or NPS 2143 (the NPS 2143 group), once a day for 2 weeks. The left untreated group was regarded as the blank control group. The dynamic growth of individual tumors in each group was monitored up to 22 days. Tumors were then excised and measured for their volumes and weights. **(A)** The representative tumor xenografts in each group. **(B)** The dynamic growth curves of tumor xenografts in each group. **(C)** The statistical comparisons of tumor volumes of the nude mice at the 22nd day (data included in figure). **(D)** The statistical comparisons of tumor weights of the nude mice at the 22nd day. Error bars stand for means ± standard deviations (SD). Significance was assessed using one-way ANOVA followed by Fisher LSD method. (*n* = 8, ^∗^*p* < 0.05 vs. control and empty vector, #*p* < 0.05 vs. shRNA-CaSR and NPS2143).

To test the role of CaSR *in vivo*, we established xenograft tumors with BALB/c nude mice. The mice were randomized into five groups (blank control, empty vector, shRNA-CaSR, cinacalcet, and NPS 2143) and inoculated them with the same quantity of untreated or treated A549 cells. The mice in the three groups were implanted with untreated A549 cells plus no additional treatment (blank control group), cinacalcet treatment (cinacalcet group), or NPS 2143 treatment (NPS 2143 group). The other two groups of BALB/c nude mice were implanted with empty vector-transfected A549 (the empty vector group) or shRNA-CaSR-transfected A549 (the shRNA-CaSR group). The growth of implanted tumors was monitored for 22 days (Figure 3A). The dynamic tumor growth rates in the shRNA-CaSR group and the NPS 2143 group were obviously faster than the blank control group, while the empty vector group remained almost unchanged. In contrast, tumors in the cinacalcet group grew much slower than those in the blank control group (Figure 3B). Individual tumor volumes and weights in each group were calculated and their statistical values were compared. The data showed that tumor volumes and weights increased prominently (^∗^*p* < 0.05, Figures 3C,D), either by decreasing CaSR protein expression (shRNA-CaSR group vs. empty vector group), or by decreasing CaSR activity (NPS 2143 group vs. blank control group). Meanwhile, increasing CaSR activity (cinacalcet group vs. blank control group) resulted in statistical reductions in both tumor volumes and weights (^∗^*p* < 0.05, Figures 3C,D). Thus, we conclude that CaSR inhibited *in vivo* LUAD growth.

### CaSR Played a Proliferation-Inhibiting Role in LUAD Possibly by Regulating the GSK3β/Cyclin D1 Pathway

**FIGURE 4:**
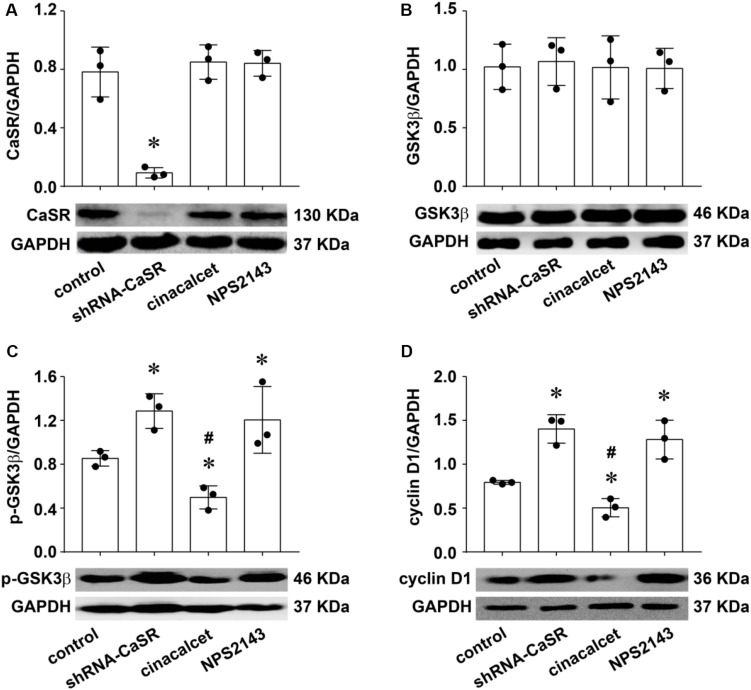
The changes of CaSR, GSK3β, p-GSK3β, and Cyclin D1 protein expression in A549 cells by shRNA-CaSR plasmid transfection, CaSR agonist, or antagonist treatment. Western blot analysis for CaSR (A,*p < 0.05 vs. control, cinacalcet, and NPS2143), GSK3β (**B)**, P-GSK3β (C,*p < 0.05 vs. control, #*p* < 0.05 vs. shRNA-CaSR and NPS2143), and Cyclin D1 (D,*p < 0.05 vs. control, #*p* < 0.05 vs. shRNA-CaSR and NPS2143) in untreated or treated A549 cells. Band densities were normalized with GAPDH (upper), which was used for evaluating equal amounts of protein loading. *n* = 3 for each. Representative Western blot assay in A549 cells of each group (lower). Significance was assessed using one-way ANOVA followed by Fisher LSD method.

A recent report suggested that CaSR suppresses the malignant behavior of colonic cancer cells by regulating the Wnt signaling pathway including GSK3β and Cyclin D1 (Aggarwal et al., 2015), which was proved to be vitally important in the development and the growth of LUAD in previous studies, including ours (Li et al., 2008; Yang et al., 2015). Meanwhile, GSK3β activity has been shown to be downstream event of CaSR (Sun and Murphy, 2010; Chiarini et al., 2017). Herein, we investigated whether the proliferation-inhibiting role of CaSR in LUAD was associated with the GSK3β/Cyclin D1 pathway. First, we changed the protein expression or the activity of CaSR by either shRNA-CaSR plasmid transfection or agonist and antagonist treatment in A549 cells, and then detected the protein expression level of CaSR, GSK3β, p-GSK3β, and Cyclin D1. As shown in Figure 4A, in contrast to the control, the CaSR protein expression level in the shRNA-CaSR-transfected group obviously decreased (^∗^*p* < 0.05), whereas both the CaSR agonist cinacalcet group and the antagonist NPS2143 group remained almost unchanged. Differing from a recent study that indicated that cinacalcet upregulated CaSR protein expression of parathyroid glands in hemodialysis patients (Sumida et al., 2013), our data suggested that in A549 cells, neither agonists nor antagonists of CaSR changed its activity by regulating its protein expression.

The GSK3β expression level remained unchanged after altering CaSR expression/activity (Figure 4B). Since the activation of GSK3β is negatively regulated by phosphorylation of serine 9 (Doble and Woodgett, 2003), the expression level of p-GSK3β (ser 9), which has no enzyme activity, could represent its activity condition (Li et al., 2008). In our study, as Figure 4C showed, inhibiting CaSR activity either by shRNA-CaSR plasmid transfection or by antagonist NPS 2143 greatly decreased GSK3β activity, demonstrated by increased levels of p-GSK3β (^∗^*p* < 0.05), whereas CaSR agonist cinacalcet exerted the opposite effect (p-GSK3β decreased, ^∗^*p* < 0.05). The results demonstrated that CaSR positively regulated GSK3β activity in LUAD A549 cells, and, in turn, the phosphorylation regulation of GSK3β by cinacalcet/NPS 2143 demonstrated their effectiveness on CaSR activation/inhibition.

Cyclin D1 is an important regulator of G1/S phase monitory point in the cell cycle. It was reported that GSK3β negatively regulated Cyclin D1 in LUAD and other tumors (Lu et al., 2008; Liao et al., 2014). Consistent with the previous studies, in the current experiment, the expression levels of Cyclin D1 in the shRNA-CaSR stable-transfection group and the NPS2143 group were statistically increased (^∗^*p* < 0.05) while their counterpart in the cinacalcet group decreased (^∗^*p* < 0.05) in contrast to the control group (Figure 4D).

Based on the above findings, we concluded that CaSR positively regulated the activity of GSK3β, with a following downregulation of Cyclin D1. Therefore, we hypothesized that the proliferation-inhibiting effect of CaSR in LUAD A549 cells was possibly via the GSK3β/Cyclin D1 pathway.

**FIGURE 5:**
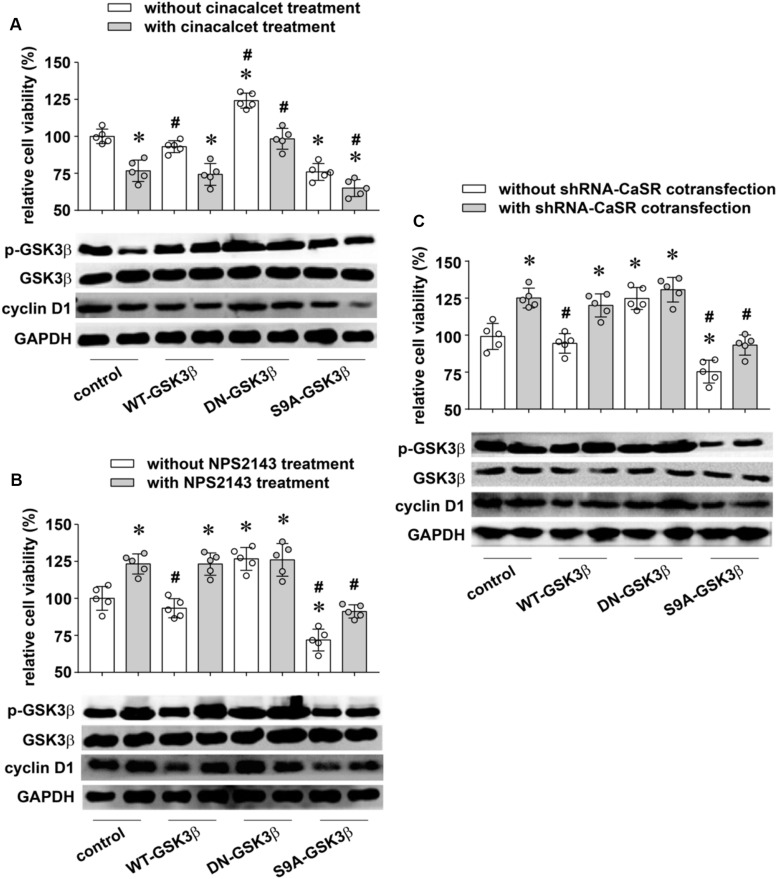
The reverse experiments for verifying the proliferation-inhibitory role of CaSR in LUAD cell A549. (**A**, upper) The effects of cinacalcet on A549 cell proliferation with or without manipulation of GSK3β activity (*p < 0.05 vs. control without cinacalcet treatment, #*p* < 0.05 vs. control with cinacalcet treatment). (**B**, upper) The effects of NPS2143 on A549 cell proliferation with or without manipulation of GSK3β activity (*p < 0.05 vs. control without shRNA-CaSR cotransfection, #*p* < 0.05 vs. control with shRNA-CaSR cotransfection). (**C**, upper) The effects of shRNA-CaSR plasmid transfection on A549 cell proliferation with or without manipulation of GSK3β activity (*p < 0.05 vs. control without NPS2143 treatment, #*p* < 0.05 vs. control with NPS2143 treatment). (**A–C**, lower) The protein expression of p-GSK3β, GSK3β, and Cyclin D1 in A549 cells of each group. GAPDH was used as a marker for equivalent amounts of protein loading. *n* = 3–5 for each experiment. Significance was assessed using one-way ANOVA followed by Fisher LSD method.

To test this hypothesis, we changed the GSK3β activity by transiently transfecting GSK3β mutant plasmids into A549 cells. On one hand, the continuously activated type S9A-GSK3β mutant overexpressed GSK3β after transfection, which led to an activation of GSK3β because of ser9 dephosphorylation (decreased p-GSK3β expression). On the other hand, the dominant negative mutant type DN-GSK3β transfection gave the opposite result, which was consistent with our previous study in A549 cells (Li et al., 2008). We then altered CaSR activity with the treatments mentioned above to monitor how CaSR changed A549 cell proliferation at different GSK3β activity levels. Our results showed that both cinacalcet treatment (activating CaSR) and S9A- GSK3β (activating GSK3β) were able to inhibit A549 cell growth, while treatment with NPS 2143 or shRNA-CaSR (decreasing CaSR activity) and DN-GSK3β transfection (decreasing GSK3β activity) both promoted A549 cell proliferation (Figures 5A–C, upper). Thus, CaSR activation and GSK3β activation produced the same effect on promoting A549 cell proliferation. Furthermore, Western blot assay revealed the expression levels of p-GSK3β and Cyclin D1 were apparently suppressed by cinacalcet treatment, but remarkably recovered by an additional treatment of DN-GSK3β (Figure 5A, lower). As we expected, the proliferation-inhibiting function of cinacalcet was suppressed by DN-GSK3β transfection (Figure 5A, upper). Simultaneously, the proliferation-promoting role of NPS 2143 treatment or shRNA-CaSR transfection was eliminated by co-treating with S9A-GSK3β transfection (Figures 5B,C, upper), which was closely associated with the changes of corresponding p-GSK3β and Cyclin D1 expression levels (Figures 5B,C, lower). These reverse experiments strongly suggested that CaSR regulated the proliferation of A549 cells, at least partially through the GSK3β/Cyclin D1 pathway.

## Discussion

Previous documents suggest that CaSR may possess possible roles as either a tumor promoter or a tumor suppressor in different organs, since CaSR regulates various pathophysiological processes, including cell proliferation, differentiation, etc. in a tissue-dependent manner (Tennakoon et al., 2016; Hannan et al., 2018; Das et al., 2020). So far, three published studies reported the function of CaSR in lung cancers. Two of them are about human lung squamous cell carcinoma (LUSCC), including a retrospective study analyzing the metastasis-related genes with 170 LUSCC patients (Dong et al., 2019), and an experimental study with LUSCC cell lines as well as related xenograft tumors (Lorch et al., 2011). The other one is a clinical study involving LUAD patients with 117 resected LUAD specimens (Wen et al., 2015). By TCGA database analysis, Dong et al. (2019) showed in LUSCC patients that the upregulation of CaSR was related to lymphatic metastasis in T1-2 stage. In the experimental study of CaSR and squamous cell carcinoma, Lorch et al. (2011) demonstrated that increasing CaSR expression/activity resulted in humoral hypercalcemia of malignancy, but did not affect tumor cell proliferation in either cultured SCC cell lines or SCC xenograft tumors. In the latter clinical study about CaSR and LUAD, Wen et al. (2015) found that a strong expression of CaSR in resected LUAD tissues was statistically related with longer overall survival periods than normal CaSR expression ones, but they did not detect the effect of CaSR on tumor cell proliferation. In this study, we firstly demonstrated the proliferation-inhibiting role of CaSR in resected LUAD specimens from the patients (Figure 1), human LUAD cell line A549 (Figure 2), and LUAD xenograft tumors (Figure 3). Our results were different from the data shown by Lorch et al. (2011) in human lung SCC, which demonstrated no effect of CaSR on SCC proliferation. From the above points, we believe that the function of CaSR is not only organ- or tissue-dependent, but also disease- or cell type-dependent. Correspondingly, the mechanisms underlying CaSR function might be disease-specific.

CaSR has been reported to adjust cell proliferation through the Wnt/β-catenin pathway in gastric cancer and colonic epithelium (Rey et al., 2012; Xie et al., 2017). Wnt pathway is correlated with the poor survival of LUAD patients since the activation of this important pathway is essential for the proliferative potential of lung cancer cells (Tammela et al., 2017). Several studies including ours have shown that the inactivation of GSK3β activated Wnt signaling and increased Cyclin D1 expression, which was regulated by the Wnt/β-catenin pathway in LUAD (Li et al., 2007, 2008; Gao et al., 2013). Meanwhile, GSK3β seems to be a downstream molecule of CaSR (Sun and Murphy, 2010; Aggarwal et al., 2015; Chiarini et al., 2017). Therefore, we then investigated if CaSR regulates LUAD cell proliferation via the GSK3β/Cyclin D1 pathway.

By changing CaSR expression/activity and then detecting GSK3β activity and Cyclin D1 protein expression, we demonstrated that CaSR positively regulated GSK3β activity in LUAD A549 cells, followed by a downregulation of Cyclin D1 expression (Figures 4, 5). Meanwhile, functional experiments showed that increasing GSK3β activity attenuated A549 cell proliferation, while decreasing GSK3β activity elicited a rapid growth, quite similar to the effects produced by increasing or decreasing CaSR activity (Figure 5). The relationship between CaSR and GSK3β in protein expression and their similar functions in cell growth suggested that GSK3β is a mediator of the proliferation-inhibiting effect of CaSR in LUAD. Moreover, the reverse experiments showed that the proliferation-inhibiting effect of CaSR was suppressed or enhanced when GSK3β activity was decreased or increased, respectively (Figure 5). Thus, our results strongly suggested that the possible mechanism of the proliferation-inhibiting effect of CaSR in LUAD was via activated GSK3β and downregulated Cyclin D1. Consistent regulation of Wnt pathway including GSK3β and Cyclin D1 by CaSR had been addressed in a previous study about colon cancer (Aggarwal et al., 2015) in which the authors observed upregulated GSK3β mRNA expression, reduced Cyclin D1, and decreased β-catenin nuclear translocation in HT29CaSR cells (colon cell line HT29 overexpressing the CaSR), and verified that the inhibition of the Wnt signaling was responsible for decreased invasive potential of HT29CaSR cells. On the contrary, in gastric cancer, CaSR activation enhanced Wnt/β-catenin signaling in tumor cells, and consequently promoted cell proliferation, migration, and invasion (Xie et al., 2017). Many other pathways were described to be downstream targets of CaSR in regulation of tumor cell proliferation. For example, in renal cell cancer, in addition to AKT signaling pathway, PLCγ-1, p38α, JNK, and PTEN, but not ERK1/2, were involved in CaSR-dependent bone metastasis and cellular proliferation (Joeckel et al., 2014). El Hiani et al. (2009) demonstrated that the proliferative effect of CaSR on breast cancer MCF-7 cells was mediated by the upregulation of TRPC1 protein and the activation of the ERK1/2 pathway. Nevertheless, continuously activated ERK1/2 by overexpressing CaSR facilitated apoptosis and reduced cell viability in neuroblastoma cell lines (Casalà et al., 2013).

Together with this research, we summarized four points about CaSR functions and their corresponding mechanisms in tumors. (i) CaSR usually possesses different capabilities in various tumors and the mechanisms differ from each other; the function of CaSR is disease-specific (El Hiani et al., 2009; Joeckel et al., 2014; Aggarwal et al., 2015; Xie et al., 2017; Iamartino et al., 2018); (ii) in different tumors, CaSR may regulate the same downstream pathway in opposite ways to generate unlike actions (Aggarwal et al., 2015; Xie et al., 2017); (iii) CaSR may modulate different downstream pathways to induce the same or similar effects in different tumors (El Hiani et al., 2009; Joeckel et al., 2014; Xie et al., 2017); and (iv) CaSR may similarly regulate the same downstream pathway, but elicit diverse or even opposite results in different tumors (El Hiani et al., 2009; Casalà et al., 2013). Facing such conflicts, it is difficult to find a perfect theory to explain all these phenomena. Notably, in the majority of the above documents, Ca^2+^ was intimately involved in the interaction with CaSR in regulating tumor behaviors such as proliferation (El Hiani et al., 2009; Joeckel et al., 2014; Aggarwal et al., 2015; Xie et al., 2017). Our preliminary experiments in LUAD A549 cells showed that CaSR induced irregular calcium oscillations under hypoxia. In previous works, we have demonstrated that irregular oscillations were able to regulate transcriptional activities of downstream genes and cellular biological behaviors via cumulative spike duration and spike amplitude (Song et al., 2012; Tan et al., 2013). Thus, we suggest that in different tumors, CaSR-mediated irregular oscillation activates different transcription factors and therefore regulates tumor cell growth or other biological behaviors in different ways.

To sum up, for the first time, we discovered the proliferation-inhibiting role of CaSR in LUAD and its possible mechanism. Because of the key role of CaSR in LUAD, it could be a potential target for future LUAD therapies. The existing CaSR agonists, such as cinacalcet, which is approved by FDA and used to treat secondary hyperparathyroidism, may present a new application to treat LUAD. However, tissue-specific delivery should be considered due to the different roles of CaSR in the different malignancies.

## Data Availability Statement

The raw data supporting the conclusions of this article will be made available by the authors, without undue reservation, to any qualified researcher.

## Ethics Statement

The studies involving human participants were reviewed and approved by Ethics Committee of Tongji Medical College, HUST. The patients/participants provided their written informed consent to participate in this study. The animal study was reviewed and approved by Ethics Committee of Tongji Medical College, HUST.

## Author Contributions

QH, JL, and LZ: conception and design of studies. PL, JL, KW, ZM, LZ, and QH: methodology and analysis. LZ, PL, and RX: drafting. JL, PL, and QH: writing. All authors contributed to the article and approved the submitted version.

## Conflict of Interest

The authors declare that the research was conducted in the absence of any commercial or financial relationships that could be construed as a potential conflict of interest.

## Abbreviations

CaSR: calcium sensing receptor
DN-GSK3 β: dominant negative GSK3 β plasmid
LUAD: lung adenocarcinoma
LUSCC: lung squamous cell carcinoma
S9A-GSK3 β: constitutively active GSK3 β plasmid
WT-GSK3 β: wild-type GSK3 β plasmid
DN-GSK3 β: dominant negative GSK3 β plasmid.
